# Effectiveness and cost-effectiveness of chiropractic and physiotherapy for chronic low back pain: a multicenter RCT in Sweden

**DOI:** 10.1186/s12891-025-08392-7

**Published:** 2025-02-25

**Authors:** Filip Gedin, Martin Skeppholm, Vibeke Sparring, Niklas Zethraeus

**Affiliations:** 1 Department of Learning, Informatics, Karolinska Institute, Management and Ethics (LIME), Nobels väg 9, D3, Stockholm, 171 77 Sweden; 2 Department of Clinical Neuroscience, Karolinska Institutet, Stockholm, Sweden; 3 Ryggkirurgiskt Centrum Stockholm AB, Sophiahemmets sjukhus, Stockholm, Sweden; 4 Stockholm Health Care Services, Stockholm, Sweden

**Keywords:** Physiotherapy, Cost-effectiveness, Low back pain, Chiropractic care, Primary health care

## Abstract

**Objective:**

To evaluate the effectiveness and cost-effectiveness of physiotherapy, chiropractic care, and the combination of physiotherapy and chiropractic care compared with information and advice for the treatment of patients with nonspecific chronic low-back pain (CLBP) in Sweden.

**Design:**

A multicentre pragmatic randomized controlled trial.

**Setting:**

Ten primary care rehabilitation units in Sweden.

**Participants:**

Eighty-eight participants with nonspecific CLBP.

**Interventions:**

The participants were randomly assigned to receive physiotherapy, chiropractic care, combination treatment, or information and advice.

**Main outcome measures:**

This study measured the Oswestry Disability Index (ODI), health-related quality of life (HRQoL), quality-adjusted life-years (QALYs), working status, and costs.

**Results:**

The study revealed no statistically significant differences in any of the outcome measures when physiotherapy, chiropractic care, and combination treatment with information and advice were compared (*p* > 0.05). The ODI changes between baseline and the 6-month follow-up ranged from 6.13 to 12.56 across the treatment groups, indicating reduced disability in all groups. Compared with the other treatment options, the combination treatment resulted in the greatest QALY gain (0.418) and lowest cost (SEK 3,081).

**Conclusion:**

Compared with alternative standalone treatment options, the combination treatment strategy resulted in greater QALY gain and lower costs from a heath care perspective. Although the study did not detect statistically significant differences in outcomes or costs among the treatment options, the combination treatment showed promising potential for cost-effectiveness. Given the small sample size and low statistical power of the study, further clinical trials with fewer treatment arms and a focus on the combination group are warranted to confirm these findings. The insights gained from this study are important for informing the design and conduct of future clinical studies investigating the effectiveness, costs and cost-effectiveness of treatments for CLBP.

**Trial registration:**

The study is registered in the ISRCTN registry (2017-02-20: ISRCTN15830360).

**Supplementary Information:**

The online version contains supplementary material available at 10.1186/s12891-025-08392-7.

## Introduction

Low back pain, which affects approximately 30% of the global population, is a significant health issue worldwide [2]. Disability due to low back pain has increased by 54% since the 1990s and is estimated to be the cause of disability in 60 million people [3]. While most individuals recover from low back pain within 6 to 12 weeks [4], a significant number develop nonspecific chronic low back pain (CLBP), leading to persistent pain, impaired function, and reduced health-related quality of life (HRQoL) [4]. Research indicates that 40–50% of back pain cases improve within the first week, and 85–90% within 6 to 12 weeks. However, this does not fully capture the long-term outlook. Studies have shown that many patients continue to experience pain over 1 to 2 years, with 62% having one or more relapses within a year and 40% still experiencing pain at 6 months. Even though 95% of patients may regain near pre-episode functionality within 6 months, 31% still report pain during activities [5]. CLBP is defined as low back pain that is not attributable to a known specific pathology, such as fractures, fibromyalgia, or tumours.

In addition to having a negative impact on people’s health, CLBP constitutes a major problem in terms of societal costs [6, 7]. It has been shown that after 12 weeks of low back pain, the recovery rate is highly uncertain; after 6 months, only 50% of patients will return to work, and after 2 years, that number is close to zero [4]. Low back pain is one of the most frequent causes of short-term or permanent marginalization from the labour market in terms of sick leave in Sweden at an estimated cost of 1 860 million EUR in 2001 [6–8]. Studies have shown that among CLBP patients with employment, 60% had at least one day of sickness absence due to low back pain during a 3-month period [6, 7]. In 2010, the cost of productivity losses for back pain Sweden was estimated to be 5 429 EUR per individual [9].

There are many different treatments for CLBP with varying degrees of evidence. Most national guidelines recommend physical activity, such as staying active, exercising or performing yoga [10]. Cognitive behavioural therapy and education are also widely recommended, as are spinal manipulation and nonsteroidal anti-inflammatory drugs [10]. However, the level of evidence on which treatment is most effective in decreasing pain and improving function among patients with CLBP is low [11]. The scientific basis to recommend one treatment over another is limited; hence, more research is needed to study the effect [12–14] and cost-effectiveness [15–17] of treatments for CLBP in clinical practice. This knowledge is central for the development of evidence-based guidelines and for assisting in resource allocation decisions on which treatments for CLBP should be recommended.

The aim of this study was to evaluate the effectiveness and cost-effectiveness of physiotherapy, chiropractic care, and the combination of physiotherapy and chiropractic care compared with information and advice in the treatment of patients with CLBP in Sweden.

## Materials and methods

### Design overview

This multicentre, pragmatic randomized controlled trial (RCT) was approved by the Regional Ethical Review Board, Stockholm (Dnr: 2016/1318-31-31), and was prospectively registered in the ISRCTN Registry (2017-02-20: ISRCTN15830360). The analysis followed a predefined study protocol published on December 22, 2017, prior to data collection completion and randomization code breaking [18]. The trial is reported according to the guidelines of reporting pragmatic trials: an extension of the Consolidated Standards of Reporting (CONSORT) statement [19]. Patient enrollment began on April 1, 2017, and ended on December 31, 2019.

### Setting and participants

The study was conducted in ten primary care rehabilitation units (PCRUs) in Sweden (nine in Region Stockholm and one in the Jönköping County Region). PCRUs are primary healthcare facilities that provide rehabilitation services to patients with various conditions, including CLBP. The participants were selected on the basis of the inclusion and exclusion criteria outlined in Table 1.

**Table 1:** Inclusion and exclusion criteria for recruitment of study participants

Inclusion	Exclusion
• Between 18–60 years • Pain located below the costal margin and above the inferior gluteal folds • Reoccurring low back pain for at least 3 months • Can stand or walk independently • Swedish speaking and literate	• Pain attributable to a known specific pathology (e.g., pain related to fractures, fibromyalgia, or tumour) • Pregnancy or less than 6 months postpartum or post weaning • Been treated for low back pain by a chiropractor and/or physiotherapist in the previous 1 month

Individuals calling the PCRUs and seeking care for low back pain by phone received verbal information about the study and were invited to participate. If the individual was willing to participate and met the eligibility criteria, a first visit was scheduled at the PCRU, and extended information about the study was sent by e-mail. During the first visit to the PCRU, the participants signed a letter of informed consent before the treatment started.

### Study treatments

During the first visit, all participants met a chiropractor and/or a physiotherapist for an initial clinical examination. The treatment duration, number of visits and content of the treatment were at the discretion of the chiropractor and/or physiotherapist. All participants were given written information on how to manage CLBP and advice about the importance of remaining active and avoiding rest, regardless of the treatment allocation [20].

All chiropractors and physiotherapists within primary care in Sweden are required to work evidence-based; otherwise, they would not be healthcare providers financed by the Stockholm region. We had no private providers, and most of the care is therefore standardized in terms of the length of the visit. The way the clinicians decided to treat the patients was determined by each clinician alone, as specified in the study protocol, which aligns with the pragmatic study design.

#### Randomization

The participants were randomized to one of four treatment groups via a computer-generated block randomization list, with participants allocated to one of four treatment arms (Table 2). The sequence was concealed from the researchers involved in enrolling and assessing participants by using sequentially numbered, opaque, sealed envelopes.

**Table 2:** Treatment alternatives

**Information and advice (advice)**: Participants were given oral advice and written information on how to manage CLBP and advice about the importance of staying active and avoiding rest. No specific exercises were provided.
**Physiotherapy**: The most common treatments used in physiotherapy in Sweden are training and exercise therapies such as stabilization training, functional training, mobility training and postural control [1].
**Chiropractic care**: The most common treatment used by chiropractors in Sweden are spinal manipulation defined as a high-velocity, low-amplitude movement at the limit of joint range that takes the joint beyond the passive range of movement. There are additional treatment alternatives that are also used, such as exercise and advice to stay active. [1].
**Chiropractic care and physiotherapy (combination)**: The treatment involves a combination of spinal manipulation (defined as a high-velocity, low-amplitude movement at the limit of joint range that takes the joint beyond the passive range of movement) and training or exercise (e.g. stabilization training, functional training, mobility training and postural control) [1].

### Data collection and outcome measures

Data were collected at baseline and at 3 and 6 months after baseline via a computer-based, self-reported patient questionnaire (see supplementary S1). The questionnaire at baseline included data on individual characteristics (age, sex, education, smoking status, physical activity, and pain duration). At baseline and at 3 and 6 months after baseline, the questionnaire collected data on outcome measures (back pain-related disability, pain intensity, general health, HRQoL and working status) and resource consumption.

The primary outcome was back pain-related disability, measured with the Oswestry Disability Index (ODI). The ODI varies between 0 and 100, with 0 indicating *no disability* and 100 *indicating complete disability*.

The secondary outcome was pain intensity, which was measured with a numeric rating scale for pain (NRS), general health (self-rated health (SRH), HRQoL measured by the EQ-5D-3 L instrument. The EQ-5D-3 L is a standardized instrument used to measure HRQoL and to calculate quality-adjusted life years (QALYs). It provides a comprehensive overview of a patient’s general health status, which complements the Oswestry Disability Index (ODI) that specifically measures disability related to low back pain. The Swedish experience-based value set was used to transform EQ-5D-3 L health states into HRQoL values, and a prescored Swedish experience-based value set was used to transform SRH severity levels into HRQoL values [21].

The costs during the 6 months after randomization were estimated for each treatment group. Direct costs included costs for pharmaceuticals, health care visits, clinical examinations, surgery, and hospital days. The indirect costs refer to the costs of changes in productivity (decreased labour production) and were estimated on the basis of the working status of each participant. Working status was estimated as the percentage of full-time work for each participant. The production value was estimated for the period from the first visit (at baseline) until 3 months and from 4 to 6 months. The average working status during the first three months (0–3 months) was assumed to be an average of the working status at baseline and 3 months. The average working status over 4–6 months was assumed to be an average of the working status at 3 and 6 months. To estimate the value of production, the average working status was multiplied by the average value of labour production of a Swedish worker (46 596 SEK/month) [22].

### Statistical analysis

The main analysis was conducted as an intention-to-treat (ITT) analysis for all participants included in the study [23]. The primary analysis was to evaluate the between-group differences in the changes in the Oswestry Disability Scale (ODI) score at 6 months. All the statistical tests were carried out at the 5% significance level (2-sided). One-way ANOVA was used to analyse the differences between groups in terms of the differences in the outcome variables at baseline and 6 months. The patterns of missing data and dropout were examined, and appropriate multiple imputations were used on the basis of the nature of the missing data.

#### Required sample size

A change of 10% points on the ODI scale is usually defined as the minimal clinically important difference (MCID). To detect a reduction of 10% points (SD of 15) in the ODI score, which agrees with the study of Davidson et al. [24], with a two-sided 5% significance level and a power of 80%, a sample size of 150 patients per group will be necessary, given an anticipated dropout rate of 20%.

### Cost-effectiveness analysis

The average number of QALYs for each treatment was based on HRQoL values derived from the EQ-5D-3 L. To estimate QALYs, we adjusted for potential differences in baseline HRQoL between the treatment groups. This was adjusted for in a regression analysis (OLS model) with QALYs as the dependent variable and three dummy variables for each treatment alternative (advice is the reference treatment) and baseline HRQoL as independent variables. The average number of QALYs over 6 months was calculated as the area under the curve over 6 months. To estimate the direct costs, the quantities of resource consumption were multiplied by the unit costs. The unit costs of pharmaceuticals were collected from the price database available at the Swedish Dental and Pharmaceutical Benefits Agency (TLV), and the unit costs for health care visits were based on prices in Region Stockholm for primary health care [25–27]. The unit costs for clinical examinations and hospital days were based on the unit costs in Region Stockholm and Region Skåne (see supplementary S2) [28]. The unit cost for spine surgery was based on Region Stockholm price adjustments for spine surgery [25]. We performed a sensitivity analysis on total direct costs over 6 months, where unit costs were changed (± 50%). All costs are estimated in 2021 SEK.

## Results

**Fig. 1:**
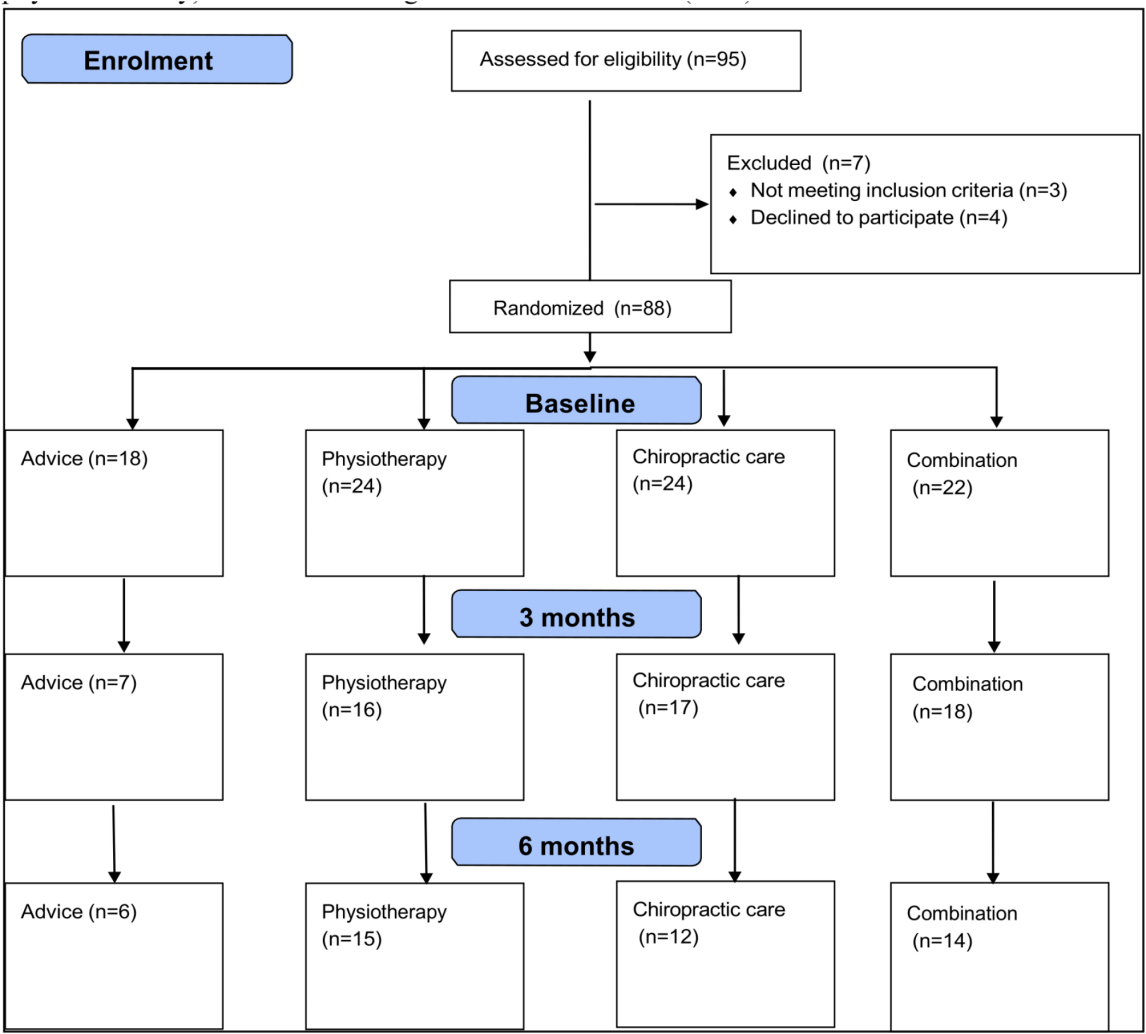
Participant flow diagram

In total, 88 participants were randomly assigned to one of four treatment groups (advice: *n* = 18; physiotherapy: *n* = 24; chiropractic care: *n* = 24; combination: *n* = 22; Fig. 1; Table 3). The dropout rate averaged 47% at the 6-month mark. Most of the 88 study participants were female (60%) and had experienced pain for more than 12 months (68%). The majority of participants were nonsmokers (89%), were physically active (75% moderate or higher physical activity) and were working at the start of the trial (90%).

**Table 3:** Baseline demographics of the study participants (values expressed as n (%) unless otherwise stated)

			Advice		Physiotherapy	Chiropractic care			Combination		
Characteristic			(*n* = 18)		(*n* = 24)	(*n* = 24)			(*n* = 22)		
Age, mean (SD)			43 (9)		46 (11)	43 (10)			45 (9)		
Sex											
	Female		14 (78)		13 (54)	12 (50)			14 (64)		
	Male		4 (22)		11 (46)	12 (50)			8 (36)		
Pain duration											
	3–12 months		3 (17)		12 (50)	6 (25)			7 (32)		
	> 12 months		15 (83)		12 (50)	18 (75)			15 (68)		
Physical activity											
	Very high		3 (17)		5 (21)	7 (29)			4 (18)		
	High		5 (28)		6 (25)	7 (29)			8 (36)		
	Moderate		7 (39)		6 (25)	8 (33)			5 (23)		
	Low		3 (17)		5 (21)	2 (8)			5 (23)		
	not at all		0		2 (8)	0			0		
Smoking											
	Yes*		2 (11)		2 (8)	3 (12)			3 (10)		
	Quit		3 (17)		8 (33)	5 (21)			6 (27)		
	Never		13 (72)		14 (58)	16 (67)			13 (59)		
Education											
	0–9 years		1 (6)		4 (17)	2 (8)			2 (9)		
	10–12 years		7 (39)		12 (50)	11 (45)			9 (41)		
	< 12 years		10 (56)		8 (33)	11 (45)			11 (50)		
ODI, mean (SD)				20.00 (12.1)	23.17 (13.1)		24.25 (11.9)		23.64 (11.4)		
NRS, mean (SD)				4.83 (2.5)	5.25 (1.7)		5.75 (2.2)		5.50 (1.8)		
EQ-5D_index,_ mean (SD)				0.82 (0.1)	0.79 (0.1)		0.79 (0.1)		0.80 (0.1)		
Working status, % (SD)				91 (25.7)	71 (37.6)		89 (25.7)		76 (34.9)		

* Yes is a combination of two alternatives: yes, daily and yes, occasionally. SD = Standard Deviation

The ODI changes between baseline and the 6-month follow-up ranged from 6.13 to 12.56 across the treatment groups, indicating reduced disability in all groups. The between-group difference in the ODI score between baseline and follow-up was not statistically significant in any of the treatment comparisons (*p* > 0.05) (Table 4).

**Table 4:** ODI score for each treatment at baseline and at 6 months

ODI Score	Advice *n* = 18	Physiotherapy *n* = 24	Chiropractic care *n* = 24	Combination *n* = 22	
	Mean (SD)	Mean (SD)	Mean (SD)	Mean (SD)	*P* value*
Baseline	20.00 (12.06)	23.17 (13.11)	24.25 (11.94)	23.64 (11.41)	0.70
3 months	15.90 (6.09)	18.78 (11.11)	18.72 (12.03)	15.38 (7.31)	0.44
6 months	9.14 (5.59)	17.04 (14.73)	11.69 (12.81)	12.06 (10.60)	0.16
Diff at 6 months	10.86 (8.14)	6.13 (11.64)	12.56 (11.10)	11.58 (14.03)	0.30

* One-way ANOVA between group comparison. SD = Standard Deviation

For pre- and postintervention measurements of the other outcome measures (numeric rating scale, self-rated health, HRQoL) between baseline and 3 and 6 months, please see supplementary material, Tables S3-S5.

The average total direct costs over 6 months varied between SEK 3 081 in the combination group and SEK 11 135 in the advice group (Table 5). There were no statistically significant differences in total direct costs between any of the treatment groups (*p* > 0.05, one-way ANOVA between group comparisons). The costs 3 months before baseline varied between SEK 1 002 and SEK 2 724 and are reported in supplementary Table S4.

**Table 5:** Mean direct cost per person (SEK) over 6 months by treatment group

	Advice		Physiotherapy	Chiropractic care	Combination
Medical visits					
	Mean (SD)		Mean (SD)	Mean (SD)	Mean (SD)
Physician		1 584 (1800)	1026 (2018)	324 (700)	234 (675)
Orthopaedic					108(424)
Nurse			304 (1200)		48 (188)
Psychologist			55 (88)	51 (110)	
Physiotherapist		542 (720)	962 (1207)	403 (789)	496 (1104)
Chiropractor		869 (1206)	248 (523)	1 420 (1280)	1247(1226)
Naprapath		239 (480)	248 (670)	25 (89)	416 (808)
Occupational Therapists				76 (305)	235 (617)
Total costs medical visits		3 235	2 843	2 299	2 784
Pharmaceuticals					
Paracetamol		59 (111)	218 (533)	81 (127)	44 (87)
Opioid			9 (36)	12 (57)	7 (26)
Ibuprofen		22 (62)	63 (99)	49 (107)	2 (10)
Ketoprofen				31 (125)	
Acetylsalicylic acid			7 (73)		29 (121)
Diclofenac		28 (56)	38 (70)	1 (7)	11 (77)
Celecoxib			12 (48)		
Total costs pharmaceuticals		109	347	174	92
Clinical examinations					
MRI		374 (834)	442 (1009)	595 (761)	204 (590)
X-ray		238 (430)			
Blood sample		179 (537)		102 (440)	
Total costs clinical examinations		791	442	697	204
Spine surgery		7 000 (17496)			
Total direct costs	11 135 (13052)		3 632 (4588)	3 170 (2671)	3 081(4015)

The value of labour production during the 6 months varied between SEK 188 714 in the physiotherapy group and SEK 220 166 in the chiropractic care group. The difference in the value of production over 6 months was not statistically significant between any of the treatment groups (Table 6) (*p* > 0.05, one-way ANOVA between-group comparison). Indirect costs based on hours of sick leave are presented in supplementary S6.

**Table 6:** Labour production (SEK) during 6 months of follow-up in the four treatment groups

	Advice		Physiotherapy	Chiropractic care		Combination	
Labour production (SEK)							
0–3 months	105 540		97 153	113 927		110 433	
4–6 months	88 066		91 561	106 239		106 239	
0–6 months	193 606		188 714	220 166		216 671	

The mean number of QALYs in each treatment group is reported in Table 7. The combination treatment had the most QALYs (0.418) during the 6-month follow-up period, followed by physiotherapy (0.414), chiropractic care (0.411), and advice (0.410). QALYs varied between 0.410 in the advice group and 0.418 in the combination group, which implies a QALY difference of 0.008 between these groups.

**Table 7:** QALYs adjusted (QALY*) and not adjusted (QALY**) for differences in baseline utility for each treatment group

Treatment	QALY* (95% CI)	QALY** (95% CI)
	(adjusted)	(unadjusted)
Advice	0.410 (0.410 to 0.431)	0.413 (0.413 to 0.434)
Physiotherapy	0.414 (0.400 to 0.430)	0.412 (0.398 to 0.428)
Chiropractic care	0.411 (0.396 to 0.426)	0.409 (0.394 to 0.424)
Combination	0.418 (0.400 to 0.434)	0.418 (0.400 to 0.434)

* QALYs during 6 months after baseline based on a regression model, adjusting for differences in baseline HRQoL. ** QALYs during 6 months after baseline without adjusting for difference in baseline HRQoL. CI = Confidence interval

Using the point estimates for the costs (direct and indirect) and QALYs (adjusted) indicates that advice and physiotherapy are dominated (associated with higher costs and fewer QALYs than the other treatments) and that combination treatment is cost-effective compared with chiropractic care given our pre-defined threshold value, or willingness to pay for a QALY, of SEK 900 000. The incremental cost-effectiveness ratio, when comparing combination treatment with chiropractic care, is SEK 850 000 (3406/0.004).

From a health care perspective, which only includes direct costs, combination treatment dominates all the other treatments (lower costs and more QALYs). These results are not sensitive to changes in the unit prices for the resources (see supplementary Table S7). Only when the price of a naprapath is increased by 50% or when the price of MRI is decreased by 50% is chiropractic care associated with the lowest costs and dominates the other treatments.

## Discussion

This multicentre pragmatic RCT revealed that, compared with standalone treatments, the combination treatment strategy resulted in a QALY gain and lower costs from a health care perspective. Although the differences in outcomes and costs among the treatment options were not statistically significant, the combination treatment showed promising potential for cost-effectiveness. This is because the combination treatment resulted in the greatest QALY gain and the lowest overall direct costs when considering all components, including pharmaceuticals, clinical examinations, and other healthcare services. These trends suggest that the combination treatment could be cost-effective compared to standalone treatments, but further research with a larger sample size is needed to confirm these findings. These encouraging trends suggest that combination treatment could lead to improved patient outcomes and more efficient use of healthcare resources. Further clinical trials with a focus on the combination group are warranted to confirm these positive findings and provide more definitive evidence.

Using the point estimate for the costs and QALYs indicates that, from a societal perspective, the combination treatment is cost-effective given a threshold value of SEK 900 000, which was the threshold value determined in our predefined study protocol [18]. Also from a health care perspective, combination treatment is cost-effective and implies lower costs and more QALYs. These results did not change if costs for surgery (only present in the advice group) were removed from the analysis, which still resulted in the direct costs for advice being highest (and costs being lowest for the combination treatment). Importantly, the nonsignificant differences in QALYs and costs between treatment groups warrant cautious interpretation of the cost-effectiveness results.

Our findings align with those of Skargren et al. (1997, 1998) [15, 29]. As in our study, Skargren did not find any statistically significant differences in costs or health outcomes at the 6-month follow-up baseline [15]. However, the direct costs in our study were slightly higher than those in Skargren et al. (1997). The difference in direct costs is partly explained by price increases (according to the consumer price index, prices increased by 32% between 1995 and 2020) [30]. In a follow-up study, Skargren et al. (1998) reported similar improvements in physiotherapy and chiropractic care groups after 12 months [15, 29].

The number of chiropractic care and physiotherapy visits was, on average, greater in the study by Skargren et al. (1997) than in our study [15]. Notably, the participants in our study also reported visits to other care providers. In the combination treatment, e.g., a participant had, on average, one visit to a naprapath, and in the chiropractic care group (where you might expect mostly chiropractor visits), the participant had, on average, one visit to a physiotherapist. This shows that individuals with CLBP seek care at a PCRU, move between different caregivers and are treated by multiple professions.

A cluster-randomized controlled trial by Saha et al. from 2019, which compared structured physiotherapy with a reference group for patients with back or neck pain in primary care, revealed no statistically significant difference in total costs between the intervention group and the reference group. They reported a statistically significant difference in QALYs when they used the Swedish value set at the 12-month follow-up. The difference in QALYs between the groups (0.033) was greater than that observed in our study (QALYs improved between 0.004 and 0.008 for the different “active” treatment groups compared with the advice group) when the physiotherapy group was compared with the advice group), suggesting that a longer follow-up period may be needed to capture the potential gain in QALYs over time [31].

### Methodological considerations

Pragmatic RCTs may be useful for assessing the cost-effectiveness of different health care programs [32, 33]. By using a pragmatic design, the results may, to a greater extent, be generalized to clinical practice. The reason is that study participants in a pragmatic trial reflect the group of patients who will be treated in clinical practice compared with study participants in a “traditional” RCT, where participants are selected on the basis of restrictive inclusion and exclusion criteria. Another strength of our study is the use of a predefined study protocol. A study protocol increases the transparency of the study methods used to collect and analyse the data and provides the opportunity for other researchers to review the study before the collection of data. Furthermore, a protocol reduces the risk of flexibility in the analysis (e.g., P-hacking) and reporting of results (e.g., selectively reporting only significant outcomes). It may also reduce the risk of publication bias, which implies that negative findings (null results) may be published to a greater extent [34].

Our study, similar to the studies by Skargren et al. (1997, 1998), is at risk of biases and limitations. The small sample size and high dropout rates reduce the statistical power and generalizability of our findings. Additionally, the pragmatic design, while reflective of real-world practice, introduces variability in treatment adherence and cross-over between groups. These factors, combined with imbalances in baseline characteristics, limit the robustness of our conclusions.

One aspect that warrants consideration in this study is related to the sample size and dropout rates. The number of participants enrolled and randomized to each group was below the calculated required sample size, and the dropout rate for the primary endpoint (6 months) was high, averaging 47%, with unbalanced dropout rates between groups. These factors undoubtedly impact the statistical power of our study and the generalizability of our findings. Unfortunately, we do not have detailed data on the specific reasons for each dropout, which is a limitation of our study. Additionally, while we planned to use adjusted analysis to control for baseline variables (age, sex, education, smoking status, physical activity, use of painkillers), the low sample size made this analysis unfeasible. Despite these limitations, our study provides valuable insights into the effectiveness and cost-effectiveness of physiotherapy, chiropractic care, and combination treatment for chronic low back pain. The pragmatic design of our trial reflects real-world clinical practice, and the findings contribute to the existing body of evidence on the management of chronic low back pain. Similar challenges have been observed in analogous primary care settings in previous research [35, 36]. For example, Bornhöft et al. [36] encountered difficulties in engaging participants through healthcare staff due to motivational constraints.

## Conclusion

The present study demonstrated that, compared with alternative standalone treatment options, the combination treatment strategy resulted in greater QALY gains and lower costs from a health care perspective. However, the study faced significant limitations, such as a small sample size, high dropout rates, and imbalances in baseline characteristics. These factors reduce the statistical power and generalizability of our findings.

Despite these limitations, the study provides valuable insights into the effectiveness and cost-effectiveness of physiotherapy, chiropractic care, and combination treatment for chronic low back pain. The pragmatic design of the trial reflects real-world clinical practice, highlighting the complexities of managing chronic low back pain and the variability in treatment adherence and cross-over between groups.

The insights gained from this study are important for informing the design and conduct of future clinical studies investigating the effectiveness, costs and cost-effectiveness of treatments for chronic low back pain. The findings underscore the need for further research with larger sample sizes, extended follow-up periods, and more rigorous methodologies. Future studies should also consider conducting feasibility studies to identify and address potential challenges prior to the definitive trial.

In conclusion, while our study has limitations, it contributes to the existing body of evidence on the management of chronic low back pain and provides a foundation for future research. This is relevant for informing clinical practice and the development of evidence-based guidelines for the treatment of chronic low back pain.

## Electronic supplementary material

Below is the link to the electronic supplementary material.

Supplementary Material 1

Supplementary Material 2

## Data Availability

Data is available upon reasonable request to the corresponding author.

## References

[1] Swedish Council on Health Technology A. Acute neck and back pain: preventive interventions – Effects of physical training, manual treatment and cognitive behavioral interventions. In. Stockholm: Swedish Agency for Health Technology Assessment and Assessment of Social Services (SBU). 2016. 28876799

[2] Vos T, Flaxman AD, Naghavi M, Lozano R, Michaud C, Ezzati M, Shibuya K, Salomon JA, Abdalla S, Aboyans V, et al. Years lived with disability (YLDs) for 1160 sequelae of 289 diseases and injuries 1990–2010: a systematic analysis for the global burden of Disease Study 2010. Lancet. 2012;380(9859):2163–96. 23245607 10.1016/S0140-6736(12)61729-2 PMC6350784

[3] GBD. DALYs, Hale collaborators: global, regional, and national disability-adjusted life-years (DALYs) for 315 diseases and injuries and healthy life expectancy (HALE), 1990–2015: a systematic analysis for the global burden of Disease Study 2015. Lancet. 2015;2016(38810053):1603–58. 10.1016/S0140-6736(16)31460-X PMC5388857 27733283

[4] Andersson GBJ. Epidemiological features of chronic low-back pain. Lancet. 1999;354(9178):581–5. 10470716 10.1016/S0140-6736(99)01312-4

[5] Triano JJ. What constitutes evidence for best practice? J Manipulative Physiol Ther. 2008;31(9):637–43. 19028247 10.1016/j.jmpt.2008.10.009

[6] Ekman M, Johnell O, Lidgren L. The economic cost of low back pain in Sweden in 2001. Acta Orthop. 2005;76(2):275–84. 16097556 10.1080/00016470510030698

[7] Ekman M, Jonhagen S, Hunsche E, Jonsson L. Burden of illness of chronic low back pain in Sweden: a cross-sectional, retrospective study in primary care setting. Spine (Phila Pa 1976). 2005;30(15):1777–85. 16094281 10.1097/01.brs.0000171911.99348.90

[8] Hansson T, Jensen I, Swedish Council on Technology Assessment in Health Care (SBU). Chapter 6. Sickness absence due to back and neck disorders. Scand J Public Health Suppl. 2004;63:109–51. 15513655 10.1080/14034950410021862

[9] Gedin F, Alexanderson K, Zethraeus N, Karampampa K. Productivity losses among people with back pain and among population-based references: a register-based study in Sweden. BMJ Open. 2020;10(8):e036638. 32792439 10.1136/bmjopen-2019-036638 PMC7430424

[10] Foster NE, Anema JR, Cherkin D, Chou R, Cohen SP, Gross DP, Ferreira PH, Fritz JM, Koes BW, Peul W, et al. Prevention and treatment of low back pain: evidence, challenges, and promising directions. Lancet. 2018;391(10137):2368–83. 29573872 10.1016/S0140-6736(18)30489-6

[11] Gedin F, Sundberg T, Sparring V, Skeppholm M, Heintz E, Zethraeus N. Umbrella Review of Primary Care treatments for adults with chronic low back Pain. J Manipulative Physiol Ther 2024;46(5-9):315-326. 10.1016/j.jmpt.2024.03.002 39297844

[12] Kamper SJ, Apeldoorn AT, Chiarotto A, Smeets RJ, Ostelo RW, Guzman J, van Tulder MW. Multidisciplinary biopsychosocial rehabilitation for chronic low back pain: Cochrane systematic review and meta-analysis. BMJ. 2015;350:h444. 25694111 10.1136/bmj.h444 PMC4353283

[13] Rubinstein SM, de Zoete A, van Middelkoop M, Assendelft WJJ, de Boer MR, van Tulder MW. Benefits and harms of spinal manipulative therapy for the treatment of chronic low back pain: systematic review and meta-analysis of randomised controlled trials. BMJ (Clinical Res ed). 2019;364:l689. 10.1136/bmj.l689 PMC6396088 30867144

[14] Saragiotto BT, Maher CG, Yamato TP, Costa LO, Costa LC, Ostelo RW, Macedo LG. Motor Control Exercise for nonspecific low back Pain: a Cochrane Review. Spine (Phila Pa 1976). 2016;41(16):1284–95. 27128390 10.1097/BRS.0000000000001645

[15] Skargren EI, Oberg BE, Carlsson PG, Gade M. Cost and effectiveness analysis of chiropractic and physiotherapy treatment for low back and neck pain. Six-month follow-up. Spine (Phila Pa 1976). 1997;22(18):2167–77. 9322328 10.1097/00007632-199709150-00015

[16] Niemisto L, Rissanen P, Sarna S, Lahtinen-Suopanki T, Lindgren KA, Hurri H. Cost-effectiveness of combined manipulation, stabilizing exercises, and physician consultation compared to physician consultation alone for chronic low back pain: a prospective randomized trial with 2-year follow-up. Spine (Phila Pa 1976). 2005;30(10):1109–15. 15897822 10.1097/01.brs.0000162569.00685.7b

[17] UKBEAM.: United Kingdom back pain exercise and manipulation (UK BEAM) randomised trial: cost effectiveness of physical treatments for back pain in primary care. BMJ 2004;329(7479):1381. 10.1136/bmj.38282.607859.AE PMC535455 15556954

[18] Gedin F, Skeppholm M, Burstrom K, Sparring V, Tessma M, Zethraeus N. Effectiveness, costs and cost-effectiveness of chiropractic care and physiotherapy compared with information and advice in the treatment of non-specific chronic low back pain: study protocol for a randomised controlled trial. Trials. 2017;18(1):613. 29273083 10.1186/s13063-017-2351-3 PMC5741874

[19] Zwarenstein M, Treweek S, Gagnier JJ, Altman DG, Tunis S, Haynes B, Oxman AD, Moher D. Improving the reporting of pragmatic trials: an extension of the CONSORT statement. BMJ. 2008;337:a2390. 19001484 10.1136/bmj.a2390 PMC3266844

[20] Jensen I. Ryggboken: en bok till dig som har ont i ryggen: baserad på de senaste medicinska rönen om ryggbesvär. Stockholm: Libris. 2004.

[21] Burstrom K, Sun S, Gerdtham UG, Henriksson M, Johannesson M, Levin LA, Zethraeus N. Swedish experience-based value sets for EQ-5D health states. Qual Life Res. 2014;23(2):431–42. 23975375 10.1007/s11136-013-0496-4 PMC3967073

[22] Avarage sallary. acording to sector, occupation, age, sex and year [http://www.statistikdatabasen.scb.se/pxweb/sv/ssd/START__AM__AM0110__AM0110A/LonYrkeAlder4/table/tableViewLayout1/?rxid=26290d43-4316-43a7-915e-ca6b2cdd0b80 ].

[23] Machin D, Campbell M, Walters S. Medical statistics. England: John Wiley & sons Ltd. 2007.

[24] Davidson M, Keating JL. A comparison of five low back disability questionnaires: reliability and responsiveness. Phys Ther. 2002;82(1):8–24. 11784274 10.1093/ptj/82.1.8

[25] Region Stockholm. Prislista vårdval primärvårdsrehabilitering inkl. Patientavgift. In. Stockholms Läns Landsting. 2020.

[26] Läkemedel. [https://www.tlv.se/beslut/sok/lakemedel/]

[27] Region Stockholm. Prisjustering inom vårdval ryggkirurgi In., vol. HSN 2017–2028;2020.

[28] Region, Skåne. Regionala priser och ersättnignar för södra sjukvårdsregionen. In.: Södra sjukvårdsregionen. 2020.

[29] Skargren EI, Carlsson PG, Oberg BE. One-year follow-up comparison of the cost and effectiveness of chiropractic and physiotherapy as primary management for back pain. Subgroup analysis, recurrence, and additional health care utilization. Spine (Phila Pa 1976). 1998;23(17):1875–83. discussion 1884. 9762745 10.1097/00007632-199809010-00016

[30] Prisomräknaren. [https://www.scb.se/hitta-statistik/sverige-i-siffror/prisomraknaren/]

[31] Saha S, Grahn B, Gerdtham UG, Stigmar K, Holmberg S, Jarl J. Structured physiotherapy including a work place intervention for patients with neck and/or back pain in primary care: an economic evaluation. Eur J Health Econ. 2019;20(2):317–27. 30171489 10.1007/s10198-018-1003-1 PMC6438933

[32] Schwartz D, Lellouch J. Explanatory and pragmatic attitudes in therapeutical trials. J Clin Epidemiol. 2009;62(5):499–505. 19348976 10.1016/j.jclinepi.2009.01.012

[33] Drummond M, Sculpher M, Torrance G, O’Brien B, Stoddart G. Methods for the Economic Evaluation of Health Care Programmes. Oxford University Press. 2015.

[34] Chalmers I, Glasziou P. Avoidable waste in the production and reporting of research evidence. Lancet. 2009;374(9683):86–9. 19525005 10.1016/S0140-6736(09)60329-9

[35] Ho CM, Thorstensson CA, Nordeman L. Physiotherapist as primary assessor for patients with suspected knee osteoarthritis in primary care-a randomised controlled pragmatic study. BMC Musculoskelet Disord. 2019;20(1):329. 31301739 10.1186/s12891-019-2690-1 PMC6626628

[36] Bornhoft L, Larsson ME, Nordeman L, Eggertsen R, Thorn J. Health effects of direct triaging to physiotherapists in primary care for patients with musculoskeletal disorders: a pragmatic randomized controlled trial. Ther Adv Musculoskelet Dis. 2019;11:1759720x19827504. 30800175 10.1177/1759720X19827504 PMC6378424

