# Supplementary material for: Effectiveness and cost-effectiveness of chiropractic and physiotherapy for chronic low back pain: a multicenter RCT in Sweden
Source: BMC Musculoskelet Disord. 2025 Feb 25;26:190. doi: 10.1186/s12891-025-08392-7 (PMC11853996; doi:10.1186/s12891-025-08392-7)
Supplement: Supplementary file 2 — Supplementary Material 2 [file 12891_2025_8392_MOESM2_ESM.docx]

S1. Questionnaire

**Part 1. Bakgrund Information**

**Gender**

- Female
- Male

**Age**

**How long have you had your lower back pain?**

- More than 3 months but less than 12 months
- More than 12 months

**How do you rate your pain today on a scale of 0 to 10?**

**How much time do you spend in a typical week on physical activities that make you warm and/or breathless?**
E.g., brisk walking, gardening, heavy housework, cycling, swimming, strength training.

- 5 hours per week or more
- More than 3 hours, but less than 5 hours per week
- Between 1 and 3 hours per week
- Less than 1 hour per week
- Not at all

**Do you smoke?**

- Yes, daily
- Yes, occasionally
- No, I have quit
- No, I have never smoked regularly

**What is your highest completed education?**

- Primary school
- High school
- University or college

**Do you have paid work as an employee or in your own business?**

- Yes
- No

**What is your current occupation?**

**Indicate your current employment rate (% of full-time).**
If you work part-time (e.g., one day a week), indicate 20. Indicate in % of full-time (0-100).
E.g., if you have a full-time job, indicate Employment rate.

**Have you been absent from work due to illness in the last 7 days?**

- Yes
- No

**How many hours have you been absent from work due to illness in the last 7 days?**

**Part 2. Your General Health Condition**

**How do you rate your general health condition?**

- Very good
- Good
- Fair
- Poor
- Very poor

**I walk without difficulty**

- I can walk but with some difficulty
- I am bedridden

**I do not need help with my daily hygiene, food, or dressing**

- I have some problems washing or dressing myself
- I cannot wash or dress myself

**I manage my main activities**

- I have some problems managing my main activities
- I cannot manage my main activities

**I have no pain or discomfort**

- I have moderate pain or discomfort
- I have severe pain or discomfort

**I am not anxious or depressed**

- I am somewhat anxious or depressed
- I am extremely anxious or depressed

**Part 3. Back Function**

**OSWESTRY - BACK FUNCTION SCALE**
The following questions are designed to give us information about how your back function affects daily life. Answer each section and mark only the one box that applies to you. We are aware that it can be difficult to choose between two closely related statements, but please only check the box that most closely matches your situation.

**PAIN INTENSITY**

- I have no pain at the moment
- I have very mild pain at the moment
- I have moderate pain at the moment
- I have fairly severe pain at the moment
- I have very severe pain at the moment
- I have unbearable pain at the moment

**PERSONAL CARE**

- I take care of myself without causing more pain
- I take care of myself, but it causes more pain
- I take care of myself, but it hurts and I have to be careful
- I need some help, but I manage most things myself
- I need help every day with most things
- I do not dress myself, I have difficulty washing myself and stay in bed

**LIFTING ABILITY**

- I can lift heavy things without pain
- I can lift heavy things, but it causes pain
- Due to pain, I cannot lift heavy things from the floor, but it is okay if they are well placed, e.g., on a table
- Due to pain, I cannot lift heavy things, but I can manage light and medium things if they are well placed
- I can only lift very light things
- I cannot lift or carry anything

**WALKING ABILITY**

- Pain does not prevent me from walking any distance
- Pain does not prevent me from walking more than 1 km
- Pain does not prevent me from walking more than 500 m
- Pain does not prevent me from walking more than 100 m
- I can only walk if I use a cane or crutches
- I mostly stay in bed and have to crawl to the toilet

**SITTING ABILITY**

- I can sit in any chair as long as I want
- I can sit in my favorite chair as long as I want
- Pain prevents me from sitting for more than 1 hour
- Pain prevents me from sitting for more than 30 minutes
- Pain prevents me from sitting for more than 10 minutes
- Pain prevents me from sitting at all

**STANDING ABILITY**

- I can stand as long as I want without pain
- I can stand as long as I want, but it causes more pain
- Pain prevents me from standing for more than 1 hour
- Pain prevents me from standing for more than 30 minutes
- Pain prevents me from standing for more than 10 minutes
- Pain prevents me from standing at all

**SLEEP**

- Pain does not prevent me from sleeping
- Pain sometimes prevents me from sleeping
- Due to pain, I sleep less than 6 hours per night
- Due to pain, I sleep less than 4 hours per night
- Due to pain, I sleep less than 2 hours per night
- Pain prevents me from sleeping at all

**SEXUAL FUNCTION**

- My sex life is normal and causes no extra pain
- My sex life is normal but causes some extra pain
- My sex life is nearly normal but very painful
- My sex life is severely restricted due to pain
- My sex life is almost non-existent due to pain
- Pain prevents me from having sex at all

**SOCIAL LIFE**

- My social life is normal and does not increase pain
- My social life is normal but increases pain
- Pain does not significantly affect my social life, but prevents me from performing more strenuous activities
- Pain has limited my social life and I do not go out as often
- Pain has limited my social life and I have to stay at home
- I have no social life due to pain

**TRAVEL**

- I can travel anywhere without pain
- I can travel anywhere but it causes pain
- Pain is severe but I can manage trips over 2 hours
- Pain is severe but I can manage trips over 1 hour
- Pain means I can only make necessary trips shorter than 30 minutes
- Pain prevents me from making any trips other than for treatment

**Part 4. Healthcare Utilization**

**Have you taken medication for your lower back pain in the last 2 weeks?**

- Yes
- No

**What type of medication do you take and how many tablets in the last 2 weeks?**
Indicate the number of tablets in the last 2 weeks.

- Paracetamol (e.g., Alvedon)
- Opioid preparations (e.g., Codeine, Tramadol, Oxynorm)
- Ibuprofen (e.g., Ipren, Brufen, Ibumetin)
- Ketoprofen (e.g., Orudis, Zon)
- Acetylsalicylic acid (e.g., Aspirin, Albyl, Dispril, Magnecyl, Treo, Bamyl)
- Diclofenac (e.g., Voltaren, Diclofenac, Eeze)
- Piroxicam (e.g., Brexidol)
- Celecoxib (e.g., Celebra)
- Etoricoxib (e.g., Arcoxia)
- Other (please specify if you checked "Other")

**Have you visited any healthcare provider for your lower back pain in the last 3 months?**
E.g., doctor, physiotherapist, chiropractor.

- Yes
- No

**Which healthcare providers have you seen and how many times have you seen each provider in the last 3 months?**
Indicate the number of visits in the last 3 months.

- General practitioner
- Orthopedic doctor
- Psychologist
- Chiropractor
- Naprapath
- Physiotherapist
- Occupational therapist
- Nurse
- Dietitian
- Other (please specify if you checked "Other")

**Have you undergone any type of medical examination for your lower back pain in the last 3 months?**
E.g., X-ray.

- Yes
- No

**Indicate which medical examination and the number of examinations you have had in the last 3 months.**
1, 2, 3, or 4 or more.

- MRI
- X-ray
- CT scan
- Ultrasound
- Blood test
- Tissue sample
- Other (please specify if you checked "Other")

**Have you been hospitalized due to your lower back pain in the last 3 months?**

- Yes
- No

**How long were you hospitalized?**
Number of days:

S2. Medical care resources and unit* costs (2020 costs in SEK)

| Resource | Unit cost | Source |
| --- | --- | --- |
| Medical visits |  |  |
| Physician | 1 800 | [24] |
| Orthopaedic | 1 800 | [24] |
| Nurse | 800 | [24] |
| Psychologist | 425 | [24] |
| Physiotherapist | 420 | [24] |
| Chiropractor | 420 | [24] |
| Naprapath | 420 | [24] |
| Occupational Therapist | 420 | [24] |
| Dietician | 420 | [24] |
| Pharmaceuticals |  |  |
| Paracetamol | 2 | [25] |
| Opioid | 1.67 | [25] |
| Ibuprofen | 1.8 | [25] |
| Ketoprofen | 2.37 | [25] |
| Acetylsalicylic acid | 2.65 | [25] |
| Diklofenac | 1.95 | [25] |
| Celecoxib | 4.50 | [25] |
| Clinical examinations |  |  |
| MRI | 1 700 | [25] |
| X-ray | 618 | [27] |
| Computed tomography | 1 648 | [27] |
| Ultra sound | 1 000 | [27] |
| Blood sample | 200 | [27] |
| Tissue sample | 4 326 | [27] |
| Spine Surgery | 50 000 | [27] |

*A unit refers to the cost for a single visit or service.

S3. Self-rated health (SRH) at baseline and at 6 months.

| SRH | Advice  n=18 | Physiotherapy  n=24 | Chiropractic care  n=24 | Combination  n=22 | P value* |
| --- | --- | --- | --- | --- | --- |
|  | Mean (SD) | Mean (SD) | Mean (SD) | Mean (SD) |  |
| Baseline | 0.89 | 0.86 | 0.89 | 0.89 | 0.76 |
| 6 months | 0.86 | 0.85 | 0.86 | 0.86 | 0.61 |
| Diff at 6 months | -0.03 | -0.01 | -0.03 | -0.3 | 0.50 |

* One-way Anova between group comparison

S4. Numeric rating Scale (NRS) score for each treatment at baseline, and at 6 months

| NRS | Advice n=18 | Physiotherapy n=24 | | Chiropractic care n=24 | | | Combination n=22 | | |  |
| --- | --- | --- | --- | --- | --- | --- | --- | --- | --- | --- |
|  | Mean (SD) | Mean (SD) | | Mean (SD) | | Mean (SD) | | | | P value* |
| Baseline | 4.83 (2.55) | 5.25 (1.72) | | 5.75 (2.23) | | 5.5 (1.81) | | | | 0.72 |
| 6 months | 2.86 (2.11) | 3.58 (2.48) | | 3.05 (1.93) | | 3.37 (1.92) | | | | 0.97 |
| Diff at 6 months | 1.97 (3.50) | 1.67 (3.22) | | 2.7 (2.74) | | 2.13 (2.42) | | | | 0.51 |
| * One-way Anova between group comparison | | |  |  |  | | |  |  | |

SD = Standard Deviation

S5. EuroQol-5 Dimension (EQ-5D)_index_ at baseline, and at 6 months by treatment groups

| EQ-5D_Index_ | | Advice n=18 | | Physiotherapy n=24 | | | | Chiropractic care  n=24 | | | | Combination n=22 | | |  | |  |
| --- | --- | --- | --- | --- | --- | --- | --- | --- | --- | --- | --- | --- | --- | --- | --- | --- | --- |
|  |  | Mean (SD) | | Mean (SD) | | | Mean (SD) | | |  | Mean (SD) | | P Value* | | |  | |
| Baseline |  | 0.815 (0.081) | | 0.786 (0.110) | | | | 0.785 (0.105) | |  | 0.798 (0.102) | | 0.78 | | |  | |
| 6 months |  | 0.837 (0.078) | | 0.846 (0.104) | | | | 0.850 (0.079) | |  | 0.844 (0.091) | | 0.33 | | |  | |
| Diff at 6 months | | | 0.022 (0.133) | | 0.060 (0.141) | | | 0.065 (0.144) | |  | 0.045 (0.104) | | | 0.06 | | |  |
| * One way Anova between group comparison  ** EQ-5Dindex score ranges between 0.34 and 0.97  SD = Standard Deviation | | | | | |  | | |  |  |  |  |  |  |  |  |  |

S6. Costs during 3 months before baseline in SEK.

| Medical visits | Advice | Combined | Chiropractic care | Physiotherapy |
| --- | --- | --- | --- | --- |
| Physician | 1 998 | 756 | 522 | 1134 |
| Orthopaedic | 0 | 90 | 108 | 234 |
| Nurse | 0 | 00 | 0 | 104 |
| Psychologist | 0 | 68 | 77 | 0 |
| Physiotherapist | 46 | 109 | 25 | 0 |
| Chiropractor | 46 | 21 | 0 | 80 |
| Naprapath | 512 | 420 | 0 | 130 |
| Occupational Therapists | 0 | 235 | 76 | 0 |
| Pharmaceuticals |  |  |  |  |
| Paracetamol | 23 | 18 | 40 | 190 |
| Ibuprofen | 0 | 1.71 | 30 | 53 |
| Ketoprofen | 0 | 0 | 7 | 0 |
| Acetylsalicylic acid | 0 | 15 | 0 | 0 |
| Diclofenac | 9 | 22 | 3 | 61 |
| Celecoxib | 0 | 0 | 0 | 24 |
| Clinical examination |  |  |  |  |
| MRI | 0 | 187 | 102 | 221 |
| X-ray | 68 | 68 | 0 | 0 |
| Blood test | 22 | 0 | 12 | 26 |
| Total cost | 2 724 | 2 011 | 1002 | 2 257 |

|  | Advice | Physiotherapy | Chiropractic care | Combination |
| --- | --- | --- | --- | --- |
| Medical visits | | | | |
| Physician + | 11927 | 4145 | 3332 | 3198 |
| Physician - | 10343 | 3119 | 3008 | 2964 |
| Orthopaedic + | 11135 | 3632 | 3170 | 3027 |
| Orthopaedic - | 11135 | 3632 | 3170 | 3027 |
| Nurse + | 11135 | 3784 | 3170 | 3105 |
| Nurse - | 11135 | 3480 | 3170 | 3057 |
| Physiotherapist + | 11406 | 4112 | 3371 | 3328 |
| Physiotherapist - | 10864 | 3151 | 2968 | 2833 |
| Chiropractor + | 11569 | 3755 | 3879 | 3704 |
| Chiropractor - | 10700 | 3508 | 2460 | 2457 |
| Naprapath + | 11254 | 3755 | 3182 | 3288 |
| Naprapath - | 11015 | 3508 | 3157 | 2873 |
| Occupational Therapists + | 11135 | 3632 | 3207 | 3198 |
| Occupational Therapists - | 11135 | 3632 | 3132 | 2963 |
| Pharmaceuticals |  |  |  |  |
| Paracetamol + | 11164 | 3740 | 3210 | 3103 |
| Paracetamol - | 11105 | 3523 | 3129 | 3058 |
| Opioid + | 11135 | 3636 | 3176 | 3084 |
| Opioid - | 11135 | 3627 | 3164 | 3077 |
| Ibuprofen + | 11146 | 3663 | 3194 | 3082 |
| Ibuprofen - | 11124 | 3600 | 3145 | 3079 |
| Ketoprofen + | 11135 | 3632 | 3185 | 3081 |
| Ketoprofen - | 11135 | 3632 | 3154 | 3081 |
| Acetylsalicylsyra + | 11135 | 3635 | 3170 | 3095 |
| Acetylsalicylsyra - | 11135 | 3628 | 3170 | 3066 |
| Diclofenac + | 11149 | 3650 | 3171 | 3086 |
| Diclofenac - | 11121 | 3613 | 3169 | 3075 |
| Medical tests/investigations |  |  |  |  |
| MRI + | 11322 | 3853 | 3467 | 3183 |
| MRI - | 10948 | 3411 | 2872 | 2979 |
| RTG + | 11254 | 3632 | 3170 | 3081 |
| RTG - | 11016 | 3632 | 3170 | 3081 |
| Blood test + | 11224 | 3632 | 3221 | 3081 |
| Blood test - | 11045 | 3632 | 3119 | 3081 |
| Surgery + | 14635 | 3632 | 3170 | 3081 |
| Surgery - | 7635 | 3632 | 3170 | 3081 |
| Without surgery | 4135 | 3632 | 3170 | 3081 |
| Total direct costs (base case) | 11135 | 3632 | 3170 | 3081 |

S7. Sensitivity analysis of the direct costs

The '+' symbol indicates a positive outcome or improvement, while the '---' symbol indicates no change or a negative outcome.

* In cases where resource consumption is zero for a specific cost type, the change in price will not impact the costs.
